# Association Between Serum Methylmalonic Acid (A Marker of Mitochondrial Dysfunction) and Serum Neurofilament Light Chains in a U.S. Population: a Cross‐Sectional Analysis From NHANES 2013–2014

**DOI:** 10.1002/brb3.70775

**Published:** 2025-08-12

**Authors:** Jun Wei, Yang Liu, Ya Li

**Affiliations:** ^1^ School of Basic Medical Sciences Jilin Medical University Jilin China; ^2^ Edinburgh Medical School: Biomedical Sciences College of Medicine and Veterinary Medicine The University of Edinburgh Edinburgh UK; ^3^ Zhejiang University School of Medicine Zhejiang University‐University of Edinburgh Institute Haining China

**Keywords:** association, NHANES, serum methylmalonic acid, serum neurofilament light chain

## Abstract

**Introduction:**

Serum neurofilament light chain (sNfL) is an emerging biomarker of neuronal damage in various neurological disorders. Methylmalonic acid (MMA) serves as a biomarker for mitochondrial dysfunction. This study aimed to investigate the relationship between MMA and sNfL.

**Methods:**

Data were obtained from the 2013–2014 National Health and Nutrition Examination Survey (NHANES). Multiple linear regression modeling was employed to confirm the association between MMA and sNfL, while smooth curve fitting was utilized to explore the potential nonlinear relationship. Subgroup analyses and interaction tests were conducted to assess the stability of the association across different subgroups.

**Results:**

This study included 2,070 participants with a mean age of 46.89 ± 15.36 years. In the fully adjusted model, each 1 nmol/L increase in serum MMA was associated with a 3.11 pg/mL increase in sNfL (95% CI: 0.91 to 5.31; p = 0.0056). Participants in the highest quartile of MMA had significantly higher sNfL levels compared to those in the lowest quartile (β = 5.09; 95% CI: 2.30 to 7.87; p = 0.0004). A nonlinear relationship was observed, with an inflection point at 5.51 nmol/L of MMA, while above the threshold, MMA was strongly associated with higher sNfL levels (β = 12.57, 95% CI: 7.14 to 17.99; p < 0.0001). Subgroup analyses further suggested stronger associations among individuals with diabetes or low vitamin B12 status (P for interaction < 0.05).

**Conclusions:**

Our study found a significant positive association between serum MMA and sNfL. Further prospective studies are warranted.

## Graphical abstract

In this cross‐sectional analysis of U.S. adults from NHANES 2013–2014, higher serum methylmalonic acid levels were positively associated with increased serum neurofilament light chain concentrations.

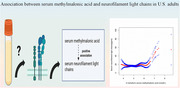

## 1 Introduction

Neurofilaments are a unique type of intermediate filaments found specifically in neurons and are made up of three main components: light, medium, and heavy chains (Petzold 2005). These structures serve as essential proteins within the neural cytoskeleton, which is critical for sustaining the structural integrity and performance of neurons (Ciardullo et al. 2023). When neuro‐axonal injury occurs, neurofilaments are discharged into the surrounding area, offering the possibility of being considered as potential biomarkers for a variety of neurological disorders (Bacioglu et al. 2016). Recent studies have revealed a notable association between the concentrations of neurofilament light chain (NfL) in cerebrospinal fluid (CSF) and serum neurofilament light chain (sNfL) (Khalil et al. 2018). This strong association has sparked heightened interest in the use of sNfL as a dependable biomarker for evaluating several neurological conditions, especially in the context of expansive epidemiological research. Additionally, earlier studies that assessed sNfL levels in a broader population context have shown a positive link between increased sNfL levels and the emergence of all‐cause dementia, Alzheimer's disease, and cognitive deterioration (De Wolf et al. 2020, Mielke et al. 2019). These results highlight the promise of sNfL both in clinical diagnostics and in advancing our understanding of the progression of neurodegenerative diseases.

Methylmalonic acid (MMA) is a diacid that plays a crucial role in the metabolic pathway related to vitamin B12, acting as a key indicator for the medical identification of vitamin B12 deficiency in humans, often proving to be more reliable than vitamin B12 itself (Kvestad et al. 2017, Doets et al. 2014). This occurs because serum concentrations of vitamin B12 can lag behind and fail to consistently reflect the intracellular levels of vitamin B12; in contrast, MMA is better suited to indicate the body's true requirement for vitamin B12 (Wang et al. 2022). Recent research indicates that MMA might possess considerable biological functions. For instance, one study revealed that the gradual buildup of MMA as individuals age can initiate transcriptional changes by increasing the expression of the SRY‐box transcription factor 4 (SOX4) gene, which may assist in tumor advancement and invasion (Gomes et al. 2020). The metabolism of the cobalamin‐associated B protein (MMAB) may adversely affect cholesterol metabolic balance due to its association with MMA (Goedeke et al. 2021). Multiple investigations have established a connection between vitamin B12 and cognitive health, with some research suggesting that serum MMA can serve as a thorough proxy for vitamin B12 levels (Boumenna et al. 2021). Importantly, heightened MMA levels have been linked with various neurological conditions (Stein et al. 2021). Consequently, the relationship between serum MMA and the level of sNfL could have significant implications for clinical research.

To our knowledge, there have been no population‐based studies that have explored the relationship between serum MMA and sNfL. Therefore, we undertook a cross‐sectional research project using data from the National Health and Nutrition Examination Survey (NHANES) to analyze the association between serum MMA levels and sNfL within a representative sample of the U.S. population.

## 2 Methods

### 2.1 Study Design and Population

In this cross‐sectional analysis, we employed publicly accessible data from the NHANES conducted during 2013–2014. The procedures and methodology for data collection utilized in NHANES are outlined in other sources (National Center for Health Statistics). In summary, NHANES is composed of a series of complex, multistage, cross‐sectional surveys carried out by the Centers for Disease Control and Prevention (CDC). These surveys aim to collect health and nutrition data from a sample of noninstitutionalized individuals within the U.S. population that is nationally representative. Initially, background information such as sociodemographics, medical history, and family history was gathered through interviews conducted in participants’ homes. Following this, participants attended a mobile examination center (MEC) to gather further essential data, including anthropometric measurements, blood pressure readings, and laboratory evaluations.

**FIGURE 1:**
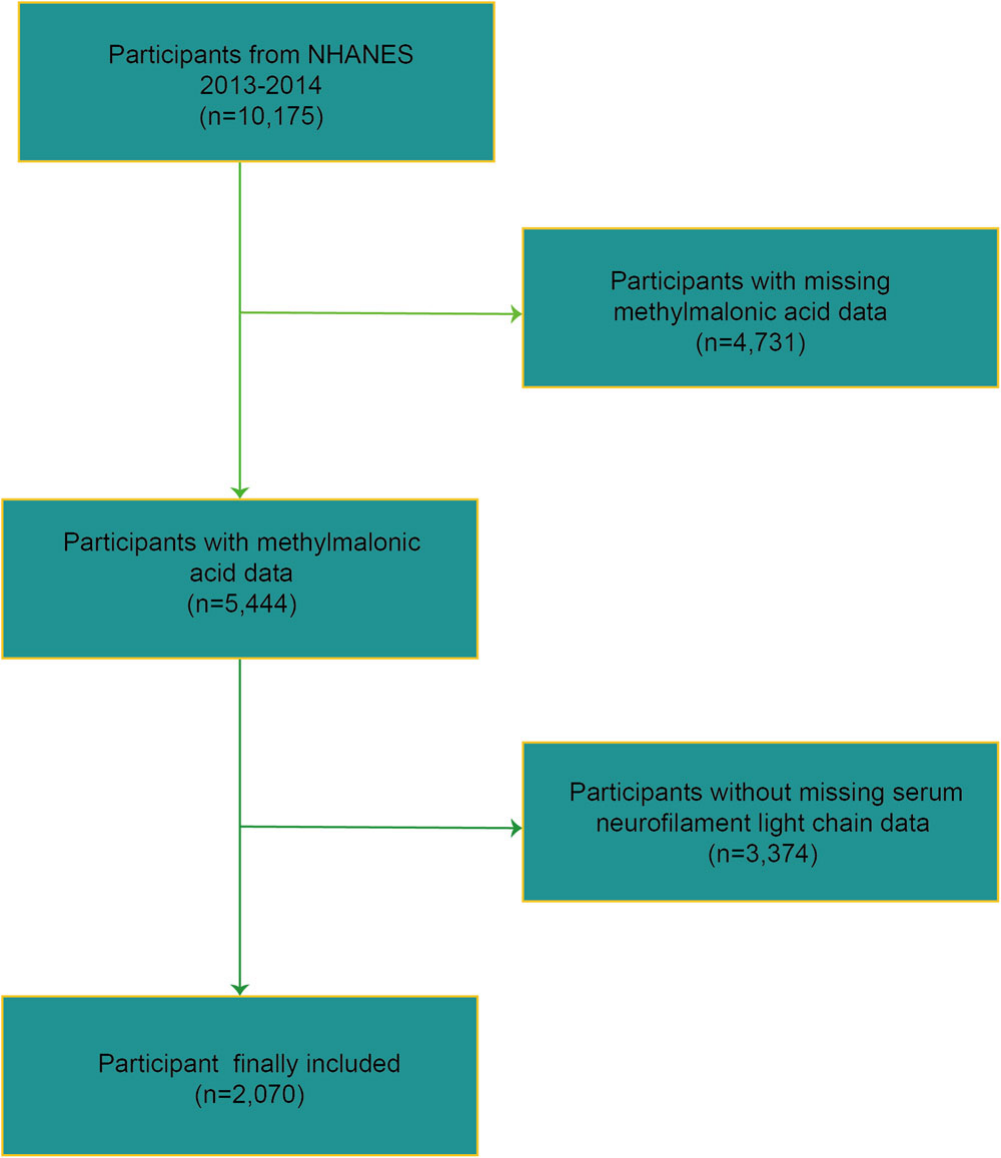
Flow chart of participants selection. NHANES: National Health and Nutrition Examination Survey.

The criteria for inclusion and exclusion in this research are depicted in Figure 1. This investigation was founded on the NHANES survey cycle of 2013–2014 because it offered complete data regarding MMA concentrations, sNfL levels, and relevant covariates. Initially, 10,175 individuals were enrolled in the 2013–2014 NHANES cycle. Of these, 4731 individuals were excluded due to missing serum MMA data, leaving 5444 participants. Among these, an additional 3374 participants were excluded due to missing sNfL data, resulting in a final sample of 2070 participants included in the present analysis. Ethical approval for NHANES was granted by the National Center for Health Statistics Research Ethics Review Board (National Center for Health Statistics, 2012).

### 2.2 Serum Methylmalonic Acid

The focus of this research was on serum MMA. To analyze serum MMA, the method used was liquid chromatography‐tandem mass spectrometry (LC‐MS/MS). Before conducting the analysis at the National Center for Environmental Health, serum samples were prepared and maintained at ‐30°C. Furthermore, the laboratory involved in the contract randomly duplicates 2% of the total sample set.

### 2.3 Serum Neurofilament Light Chain Measurement

NfL analyses were performed using a fully automated Attelica immunoassay platform, which incorporated a highly sensitive and high‐throughput acridinium‐ester (AE) immunoassay. Initially, samples underwent incubation with an AE‐conjugated antibody that specifically targeted the NfL antigen. Following this step, paramagnetic particles (PMP) coated with the capture antibody were added to the mixture, resulting in the formation of an antigenic complex associated with both the AE‐conjugated antibody and the PMP. The surplus AE‐conjugated antibody was subsequently separated and eliminated, after which acid and base were introduced to trigger chemiluminescence for the detection of light emissions. The assay's lower limit of quantification (LLOQ) was established at 3.9 pg/mL based on replication testing of low‐concentration NfL samples, while the upper limit of quantification (ULOQ) was determined to be 500 pg/mL. The LLOQ was defined as the concentration at which the coefficient of variation (CV) was 20% or less.

### 2.4 Covariate

Covariate data were collected through questionnaires, physical examinations, and laboratory tests. The following covariates were included: sex, age, race, and ethnicity, education level, marital status, smoking status, alcohol consumption, body mass index (BMI) in kg/m^2^, various ranges of vitamin B12 (pg/mL), diabetes, hypertension, the ratio of family income to poverty (PIR), alanine aminotransferase (ALT, U/L), aspartate aminotransferase (AST, U/L), total cholesterol (TC, mg/dL), triglycerides (TG, mg/dL), uric acid (UA, mg/dL), blood urea nitrogen (BUN, mg/dL), gamma glutamyl transferase (GGT, U/L), high‐density lipoprotein (HDL, mg/dL), and creatine phosphokinase (CPK, IU/L). In NHANES, self‐reported race and ethnicity information was derived from responses to survey questions regarding race and Hispanic origin. Accordingly, we categorized participants into three groups based on race and ethnicity: non‐Hispanic white, non‐Hispanic black, and other (including multiracial). Vitamin B12 levels were classified into three categories based on the following ranges: < 300 pg/mL, 300–900 pg/mL, and ≥ 900 pg/mL. Educational attainment was assessed using the questionnaire “What is the highest grade or level completed or degree earned?” for adults aged 20 years and older. Based on the responses, we categorized educational attainment into three levels: less than high school, high school or GED, and above high school. Marital status was classified into three groups: married or living with a partner, never married, and widowed, divorced, or separated. Participants who responded “yes” to the question “Have you ever smoked at least 100 cigarettes in your life?” were categorized as having a history of smoking. Additionally, individuals who answered “yes” to the question “Have you had at least 12 alcoholic drinks in the past year?” were defined as drinkers. Those who answered “yes” or “borderline” to the question “Have you ever been told by a doctor that you have diabetes, except during pregnancy?” were categorized as having a history of diabetes. The remaining biochemical indicators were derived from the ‘Standard Biochemistry Profile’. During the physical examination, the height and weight of the subjects were recorded, and body mass index (BMI) was subsequently calculated and categorized as < 25, 25–30, and ≥ 30. A questionnaire focused on medical conditions was utilized to assess the frequency of strokes. Participants were deemed to have had a stroke if they answered “yes” to the following query: “Have you ever been informed by a doctor or another healthcare professional that you had a stroke?” Prior studies have confirmed the reliability of self‐reported stroke data.

We considered the following biochemical markers in our analysis: alanine aminotransferase (ALT, U/L), aspartate aminotransferase (AST, U/L), total cholesterol (TC, mg/dL), triglycerides (TG, mg/dL), uric acid (UA, mg/dL), blood urea nitrogen (BUN, mg/dL), gamma glutamyl transferase (GGT, U/L), high‐density lipoprotein (HDL, mg/dL), and creatine phosphokinase (CPK, IU/L). These markers were selected based on their clinical relevance to metabolic and organ function.

However, ALT, AST, CPK, and GGT were not included as covariates in the final regression models because they are closely related to methylmalonic acid (MMA) in physiological and biochemical pathways. These enzymes reflect liver, muscle, and mitochondrial function and may act as parallel indicators or mediators rather than independent confounders. To avoid potential overadjustment bias, we did not adjust for them in the multivariable models.

### 2.5 Statistical Analysis

In accordance with the NHANES guidelines, we accounted for both complex sampling designs and sampling weights in our analysis of NHANES data (Zhang et al. 2024). We collected data on the concentration and distribution of MMA in serum. Normally distributed continuous variables are presented as mean ± SD, while skewed continuous variables are reported as median (IQR). Categorical variables are described by the count of observations in each category (absolute values) along with their respective proportions (percentages). To assess the association between serum MMA and sNfL levels, we employed multiple linear regression models. Three models were constructed:Model 1 was unadjusted, while Model 2 was adjusted for sex, age, and race/ethnicity. Model 3 included adjustments for sex, age, race/ethnicity, PIR, education level, diabetes, hypertension, smoking status, BMI, and vitamin B12. These models were constructed to control for potential confounding and to estimate both crude and adjusted associations. Moreover, covariates with less than 5% missing values were retained without imputation, and complete case analysis was applied.

To investigate potential nonlinear associations between serum MMA and sNfL, we applied smooth curve fitting based on generalized additive models. If a nonlinear pattern was detected, a two‐piecewise linear regression model was subsequently fitted to identify a threshold effect.

Additionally, we conducted subgroup analyses to assess the robustness of our findings across predefined strata, including sex, age, BMI, diabetes status, hypertension, and vitamin B12 levels. Interaction terms were tested to identify potential effect modification. All statistical analyses were carried out using R (version 4.2) and Empowerstats (version 5.0). It was deemed statistically significant when p < 0.05 (two‐sided).

## 3 Results

### 3.1 Participants and Demographic Baseline Characteristics

Baseline demographic characteristics of eligible subjects are shown in Table 1. Of the 2070 individuals in this study, 47.78% were male and 52.22% were female. The participants' ages ranged from 20 to 79 years, with a mean age of 46.89 ± 15.36 years. The weighted characteristics of the subjects were categorized into quartiles based on MMA levels, revealing significant differences among the quartiles concerning age, race, marital status, hypertension, smoking, vitamin B12, BUN, UA, and sNfL. Individuals in the bottom quartile were predominantly male, non‐Hispanic white, and aged 60 years or older. In comparison to other groups, participants in the bottom quartile were more likely to be unmarried and exhibited higher levels of vitamin B12 when it was within the elevated range. Conversely, being divorced, widowed, or separated, as well as having diabetes mellitus, hypertension, smoking habits, and lower vitamin B12 levels, were associated with the lower level range of these variables. Additionally, BUN and UA levels were found to be lower in this group (p < 0.05). Participants in the lowest MMA quartile (Q1) were predominantly younger, with 85.24% under 60 years old, while the proportion of individuals aged 60 or older increased progressively across quartiles, reaching 39.85% in Q4 (p < 0.001). Racial distribution also varied significantly (p < 0.001), with the proportion of non‐Hispanic White individuals increasing from 23.82% in Q1 to 54.21% in Q4. Conversely, non‐Hispanic Black participants were most prevalent in Q1 (25.59%) but declined to 13.60% in Q4, while the “Other Races” category showed a decreasing trend from 50.59% in Q1 to 32.18% in Q4. Compared to higher MMA quartiles, participants in Q1 were significantly more likely to be unmarried (p < 0.05) and had higher levels of vitamin B12 within the elevated range (p < 0.05). Conversely, Q1 participants were significantly more likely to be divorced, widowed, or separated (p < 0.05) and had a higher prevalence of diabetes mellitus (p < 0.05), hypertension (p < 0.05), and smoking habits (p < 0.05). In addition, Q1 participants exhibited significantly lower BUN and UA levels compared to higher MMA quartiles (p < 0.05). The incidence of stroke was notably lower in Q1, with only 0.79% of participants reporting a history of stroke, compared to 1.95% to 4.02% in the higher MMA quartiles (p = 0.005).

**TABLE 1:** Baseline characteristics of the study participants.

Characteristics	Total (n = 2070)	Quartile 1 (n = 508)	Quartile 2 (n = 526)	Quartile 3 (n = 514)	Quartile 4 (n = 522)	*p* value
Sex, n (%)						0.040
Male	989 (47.78%)	216 (42.52%)	256 (48.67%)	263 (51.17%)	254 (48.66%)	
Female	1081 (52.22%)	292 (57.48%)	270 (51.33%)	251 (48.83%)	268 (51.34%)	
Age, years						< 0.001
< 60	1528 (73.82%)	433 (85.24%)	399 (75.86%)	382 (74.32%)	314 (60.15%)	
≥ 60	542 (26.18%)	75 (14.76%)	127 (24.14%)	132 (25.68%)	208 (39.85%)	
Race and ethnicity, n (%)						< 0.001
Non‐Hispanic white	910 (43.96%)	121 (23.82%)	235 (44.68%)	271 (52.72%)	283 (54.21%)	
Non‐Hispanic black	373 (18.02%)	130 (25.59%)	95 (18.06%)	77 (14.98%)	71 (13.60%)	
Other Races	787 (38.02%)	257 (50.59%)	196 (37.26%)	166 (32.30%)	168 (32.18%)	
Education level, n (%)						0.247
Less than high school	454 (21.93%)	127 (25.00%)	105 (20.15%)	98 (19.07%)	123 (23.56%)	
High school or GED	431 (20.82%)	105 (20.67%)	109 (20.76%)	106 (20.62%)	111 (21.26%)	
Above high school	1185 (57.25%)	276 (54.33%)	311 (59.13%)	310 (60.31%)	288 (55.17%)	
Marital status, n (%)						< 0.001
Marriage/living with partner	1289 (62.27%)	315 (62.01%)	326 (61.98%)	337 (65.56%)	311 (59.58%)	
Never married	395 (19.08%)	124 (24.41%)	110 (20.91%)	89 (17.32%)	72 (13.79%)	
Divorced/widowed/separated	386 (18.65%	69 (13.58%)	90 (17.11%)	88 (17.12%)	139 (26.63%)	
Diabetes, n (%)						0.018
Yes	221 (10.68%)	46 (9.06%)	51 (9.70%)	49 (9.53%)	75 (14.37%)	
No	1849 (89.32%)	462 (90.94%)	475 (90.30%)	465 (90.47%)	447 (85.63%)	
Hypertension, n (%)						< 0.001
Yes	734 (35.49%)	140 (27.56%)	167 (31.75%)	197 (38.40%)	230 (44.15%)	
No	1334 (64.51%)	368 (72.44%)	359 (68.25%)	316 (61.60%)	291 (55.85%)	
Missing	2(0.10%)	0(0.00%)	0(0.00%)	1(0.19%)	1(0.19%)	
Smoking status, n (%)						< 0.001
Yes	915 (44.22%)	184 (36.22%)	220 (41.83%)	252 (49.03%)	259 (49.71%)	
No	1154 (55.78%)	324 (63.78%)	306 (58.17%)	262 (50.97%)	262 (50.29%)	
Missing	1(0.05%)	0(0.00%)	0(0.00%)	0(0.00%)	1(0.19%)	
Stroke						0.005
Yes	52 (2.51%)	4 (0.79%)	17 (3.23%)	10 (1.95%)	21 (4.02%)	
No	2018 (97.49%)	504 (99.21%)	509 (96.77%)	504 (98.05%)	501 (95.98%)	
BMI, n (%)						0.435
< 25	625 (30.43%)	150 (29.76%)	163 (31.23%)	147 (28.77%)	165 (31.91%)	
25–30	654 (31.84%)	147 (29.17%)	164 (31.42%)	179 (35.03%)	164 (31.72%)	
≥ 30	775 (37.73%)	207 (41.07%)	195 (37.36%)	185 (36.20%)	188 (36.36%)	
Vitamin B12, n (%)						< 0.001
< 300 pg/mL	227 (10.98%)	25 (4.94%)	34 (6.46%)	41 (7.98%)	127 (24.33%)	
300–900 pg/mL	1601 (77.42%)	397 (78.46%)	424 (80.61%)	419 (81.52%)	361 (69.16%)	
≥ 900 pg/mL	240 (11.61%)	84 (16.60%)	68 (12.93%)	54 (10.51%)	34 (6.51%)	
ALT, U/L	25.14 ± 17.87	23.99 ± 13.24	25.85 ± 20.74	25.70 ± 19.99	24.99 ± 16.39	0.326
AST, U/L	25.49 ± 24.62	24.29 ± 17.37	26.95 ± 39.97	25.05 ± 17.34	25.60 ± 14.34	0.359
CPK, IU/L	146.37 ± 182.25	142.02 ±140.12	156.57 ±253.32	150.25 ±177.27	136.58 ± 130.81	0.300
GGT, U/L	28.77 ± 37.95	26.20 ± 30.09	29.50 ± 42.54	28.19 ± 38.69	31.09 ± 39.10	0.205
BUN, mg/dL	12.88 ± 5.58	10.95 ± 3.52	12.30 ± 4.13	13.15 ± 3.96	15.08 ± 8.36	< 0.001
TC, mg/dL	189.23 ± 40.82	188.23 ± 41.80	187.88 ± 40.03	189.73 ± 40.72	191.09 ± 40.77	0.565
TG, mg/dL	122.40 ± 130.46	121.09 ± 202.58	113.77 ± 81.95	120.95 ± 102.99	133.81 ± 101.38	0.093
HDL, mg/dL	53.54 ± 15.80	54.30 ±15.25	53.65 ±15.96	53.62 ±15.72	52.61 ±16.25	0.386
UA, mg/dL	5.44 ± 1.40	5.16 ± 1.34	5.40 ± 1.33	5.53 ± 1.35	5.67 ± 1.53	< 0.001
sNfL, pg/ml	16.92 ± 20.44	13.18 ± 11.72	16.37 ± 25.38	15.05 ± 10.28	22.95 ± 26.82	< 0.001

Mean ± SD for continuous variables: the *p* value was calculated by the weighted linear regression model; (%) for categorical variables: the *p* value was calculated by the weighted chi‐square test. Abbreviation: MMA, methylmalonic acid; BMI, body mass index; PIR, the ratio of family income to poverty; ALT, alanine aminotransferase; AST, aspartate aminotransferase; TC, total cholesterol; TG, triglycerides; UA, uric acid; BUN, blood urea nitrogen; GGT, gamma‐glutamyl transferase; CPK, creatine phosphokinase; sNfL, serum neurofilament light chains.

### 3.2 Association Between Serum MMA and sNfL

Table 2 presents the results of the multiple linear regression model examining the relationship between MMA (µg/L) and sNfL (pg/mL). In the crude model (Model 1), MMA showed a significant positive association with sNfL levels (β = 10.22, 95% CI: 8.35 to 12.10; p < 0.0001). This association remained statistically significant after adjustment for covariates in Model 3 (β = 3.11, 95% CI: 0.91 to 5.31; p = 0.0056). When MMA was divided into four groups based on its distribution, participants in the highest group (Q4) had significantly higher sNfL concentrations compared to those in the lowest group (Q1) in Model 3 (β = 5.09, 95% CI: 2.30 to 7.87; p = 0.0004). The second (Q2) and third (Q3) groups did not show statistically significant differences compared to Q1 in the fully adjusted model.

**TABLE 2:** Associations between serum MMA and sNfL in the multiple regression mode.(N = 2067).

Variable	In MMA(nmol/L)	Quartiles of MMA levels			
		Q1	Q2	Q3	Q4
	β(95%CI), *p* value	β(95%CI), *p* value	β(95%CI), *p* value	β(95%CI), *p* value	β(95%CI), *p* value
Model 1	10.22 (8.35, 12.10) < 0.0001	Reference	3.18 (0.73, 5.64) 0.0111	1.87 (‐0.60, 4.34) 0.1380	9.77 (7.31, 12.23) < 0.0001
Model 2	7.24 (5.31, 9.17) < 0.0001	Reference	1.60 (‐0.82, 4.03) 0.1946	−0.46(−2.93, 2.01) 0.7158	5.49 (2.95, 8.02) < 0.0001
Model 3	3.11 (0.91, 5.31) 0.0056	Reference	2.03 (‐0.62, 4.67) 0.13330	−0.50(−3.16, 2.16) 0.7120	5.09 (2.30, 7.87) 0.0004

Model 1: No adjustment for any covariates. Model 2: Adjusted for sex, age, race and ethnicity. Model 3: Adjusted for sex, age, race, education level, marital status, diabetes, hypertension, smoking status, bmi, vitamin B12.

**Abbreviations**: MMA, methylmalonic acid; BMI, body mass index; sNfL, serum neurofilament light chains.

**FIGURE 2:**
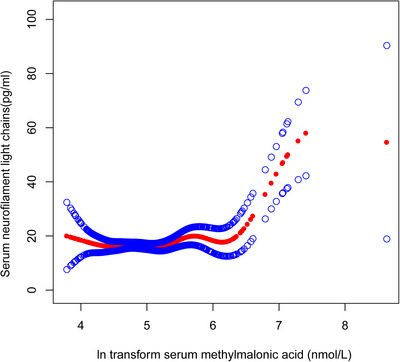
The nonlinear associations between serum MMA and sNfL. The solid red line represents the smooth curve fit between variables. Blue bands represent the 95% of confidence interval from the fit.

To further explore the potential nonlinear relationship between MMA and sNfL, smooth curve fitting was applied (Figure 2), revealing a positive nonlinear trend indicative of a possible threshold effect. To better quantify this pattern, we performed a threshold effect analysis using a two‐piecewise linear regression model (Table 3). An inflection point was identified at 5.51 nmol/L of serum MMA. Below this threshold, MMA was not significantly associated with sNfL (β = ‐0.31, 95% CI: ‐3.13 to 2.52; p = 0.8321). In contrast, above the threshold, MMA was strongly and positively associated with sNfL (β = 12.57, 95% CI: 7.14 to 17.99; p < 0.0001). The log‐likelihood ratio test comparing the piecewise model to a single‐line model showed a statistically significant improvement in model fit (p < 0.001).

**TABLE 3:** Threshold effect analysis.

sNfL	Adjusted OR(95%CI) *p* value
Total	
Standard linear model	3.11 (0.91, 5.31) 0.0056
Two‐piecewise linear model	
Inflection point	5.51
Serum MMA < 5.51 (nmol/L)	−0.31 (−3.13, 2.52) 0.8321
Serum MMA > 5.51 (nmol/L)	12.57 (7.14, 17.99) < 0.0001
Log likelihood ratio	< 0.001

The threshold effect between serum MMA and sNfL was evaluated using a two‐piecewise linear regression model. The log‐likelihood ratio test indicated a significantly better fit of the two‐piecewise model compared to the standard linear model (p < 0.001). The model was adjusted for sex, age, race, education level, marital status, diabetes, hypertension, smoking status, bmi, vitamin B12.

### 3.3 Subgroup Analyses

In subgroup analyses, we examined the effects of various variables on the relationship between MMA and sNfL levels. As illustrated in Table 4, the association between MMA and sNfL levels remained consistent across multiple subgroups, including sex, age, BMI, hypertension, smoking status, and alcohol consumption. However, the impact of MMA on sNfL exhibited significant variation in the diabetes and vitamin B12 subgroups (interaction p < 0.0001). In the presence of diabetes, each 1 µg/L increase in MMA was associated with a 0.07 unit increase in sNfL levels (p < 0.0001). Conversely, when diabetes was absent, each 1 µg/L increase in MMA corresponded to a 0.01 unit increase in sNfL levels (p = 0.0080). In the context of high vitamin B12 levels, there was a notable increase in sNfL of 0.12 pg/mL for each 1 µg/L increase in MMA. In contrast, when vitamin B12 levels were low, there was a significant increase in sNfL of 0.01 pg/mL for each 1 µg/L increase in MMA. When vitamin B12 was within the reference range, each 1 µg/L increase in MMA was associated with a significant increase in sNfL of 0.02 pg/mL. These findings suggest that both diabetes and vitamin B12 levels play significant roles in modifying the relationship between MMA and neuronal damage.

**TABLE 4:** Subgroup analysis of the association between serum MMA and sNfL.

Characteristics	β(95%CI), *p* value	P for interaction
Sex, n (%)		0.3041
Male	0.01 (0.00, 0.02), 0.0014	
Female	0.02 (0.01, 0.03), 0.0028	
Age, years		0.8442
< 60	0.01 (0.01, 0.02), 0.0001	
≥ 60	0.01 (0.00, 0.02), 0.0240	
BMI, n (%)		0.1327
< 25	0.02 (0.01, 0.04), 0.0001	
25–30	0.01 (0.00, 0.02), 0.0021	
≥ 30	0.01 (‐0.01, 0.02), 0.3444	
Diabetes, n (%)		< 0.0001
Yes	0.07 (0.05, 0.09), < 0.0001	
No	0.01 (0.00, 0.01), 0.0080	
Hypertension, n (%)		0.1704
Yes	0.01 (0.00, 0.02), 0.0010	
No	0.00 (‐0.01, 0.01), 0.8215	
Smoking status, n (%)		0.2820
Yes	0.00 (‐0.01, 0.02), 0.5979	
No	0.01 (0.01, 0.02), < 0.0001	
Alcohol		0.7250
Yes	0.01 (0.00, 0.02), 0.0356	
No	0.01 (0.00, 0.02), 0.0105	
Vitamin B12, n (%)		< 0.0001
< 300 pg/mL	0.01 (0.00, 0.02), 0.0071	
300–900 pg/mL	0.02 (0.00, 0.03), 0.0054	
≥ 900 pg/mL	0.12 (0.09, 0.16), < 0.0001	

Sex, age, race, pir, education level, marital status, diabetes, hypertension, smoking status, bmi, vitamin b12 was adjusted.

**Abbreviations**: MMA, methylmalonic acid; BMI, body mass index; sNfL, serum neurofilament light chains.

## 4 Discussion

This study demonstrates a significant positive association between serum MMA and sNfL. In the fully adjusted linear regression model, each 1 nmol/L increase in MMA corresponded to a 3.11 pg/mL increase in sNfL (95% CI: 0.91 to 5.31; p = 0.0056). Moreover, participants in the highest quartile of MMA exhibited significantly higher sNfL levels compared to those in the lowest quartile (β = 5.09; 95% CI: 2.30 to 7.87; p = 0.0004). Subgroup analyses indicated that the strength of this association varied by diabetes status and vitamin B12 levels. A nonlinear relationship was also observed, with an inflection point at 5.51 nmol/L of MMA. Above this threshold, the association between MMA and sNfL was notably stronger, whereas no significant association was detected below the threshold.

The significance of sNfL as a biomarker for neuronal injury is extensively documented in scientific literature. It has been established that sNfL levels correlate with the severity and progression of various neurodegenerative disorders, such as Alzheimer's disease, multiple sclerosis, and amyotrophic lateral sclerosis (Khalil et al. 2018, Disanto et al. 2017). Furthermore, heightened sNfL concentrations have been linked to cognitive decline within general population studies (Darmanthé et al. 2021). Nevertheless, the connection between sNfL and metabolic indicators like MMA remains insufficiently investigated. Traditionally, MMA has been evaluated as an indicator of vitamin B12 deficiency, a condition associated with disrupted DNA synthesis, myelin degradation, and neuronal impairment (Chia et al. 2005). Increased levels of MMA have been associated with cognitive decline and a heightened risk of dementia (Serrano‐Pozo et al. 2021). Our research expands on this existing knowledge by revealing a direct association between MMA and sNfL, effectively connecting metabolic dysfunction to neurodegenerative processes. In contrast to previous studies that concentrated on small, disease‐specific groups, this investigation employs a large, representative dataset from the NNHANES database. This expanded perspective improves the applicability of the results and closes a significant gap in understanding the relationship between MMA and sNfL in a broader population context.

The interactions identified between MMA and sNfL in subgroups characterized by diabetes and differing vitamin B12 levels warrant additional exploration. It is well‐established that diabetes can amplify oxidative stress and inflammation, potentially leading to increased neuronal damage (Eid et al. 2023). Our results indicate that those with diabetes are especially susceptible to rises in sNfL associated with MMA. This aligns with other research highlighting that metabolic disorders can hasten neurodegenerative changes. The differences noted in the relationships between MMA and sNfL across various vitamin B12 classifications are particularly compelling (Lin and Beal 2006). Higher vitamin B12 levels correlated with more pronounced sNfL responses to MMA, which could indicate the presence of inactive or non‐functional B12 forms commonly seen in chronic illness (Green and Miller 2022). These results emphasize the intricate nature of vitamin B12 metabolism and its effects on neuronal well‐being.

The relationship between MMA and sNfL probably encompasses mitochondrial impairment and oxidative stress. An increase in MMA interferes with energy metabolism and stimulates the generation of reactive oxygen species, resulting in neuronal damage (Tejero et al. 2024). High concentrations of sNfL, conversely, indicate axonal injury, which could stem from both immediate metabolic stress and subsequent inflammatory reactions (Taylor et al. 2022). Grasping these mechanisms is essential for creating targeted strategies to alleviate neurodegeneration.

This research showcases several significant strengths. To begin with, the large sample size contributes to the reliability of the findings. In addition, we addressed various confounding factors through multiple regression analysis, thereby reducing bias and producing more dependable results. Furthermore, we performed stratified analyses to explore the relationships among different subgroups more thoroughly. Nevertheless, care should be taken when interpreting these results due to certain limitations. Firstly, the cross‐sectional nature of the study restricts our capacity to infer causal relationships. Secondly, the variables gathered through questionnaires might not have been classified accurately. Thirdly, there could be biases in the testing methods and processes for serum MMA, which might affect the validity of the exposure variables. Finally, undiscovered confounders might result in misclassification of causality, risk assessment bias, limited applicability of results, and inaccuracies in the estimation of the association's strength between variables. Consequently, future investigations should prioritize prospective studies involving larger sample sizes or multicenter cohort research in this area. To validate the outcomes of these studies, it may be essential to include other potential confounders, apply standardized measurement techniques, and guarantee a high level of accuracy in the testing procedures.

## 5 Conclusions

Our study found a significant positive association between serum MMA and sNfL. Further prospective studies are warranted.

## Author Contributions

**Jun Wei**: conceptualization, methodology, investigation, software, data curation, writing – original draft. **Yang Liu**: investigation, validation, formal analysis, supervision, writing – review and editing. **Ya Li**: funding acquisition, visualization, project administration, resources, supervision, writing – review and editing.

## Ethics Statement

The portions of this study involving human participants, human materials, or human data were conducted in accordance with the Declaration of Helsinki and were approved by the NCHS Ethics Review Board. The patients/participants provided their written informed consent to participate in this study.

## Peer Review

The peer review history for this article is available at https://publons.com/publon/10.1002/brb3.70775.

## Data Availability

The survey data are publicly available on the internet for data users and researchers throughout the world (www.cdc.gov/nchs/nhanes/).
